# Bleb morphology following mitomycin-C sponge versus subconjunctival injection in deep sclerectomy for pediatric congenital glaucoma: A case report

**DOI:** 10.1016/j.ijscr.2025.111976

**Published:** 2025-09-22

**Authors:** Dania A. Bamefleh, Mohammed A. Halawani, Saad Alosaimi, Raghad Alonazi

**Affiliations:** a Glaucoma Division, King Khaled Eye Specialist Hospital & Research Centre, Riyadh, Saudi Arabia; b Ministry of Health, Riyadh, Saudi Arabia; c College of Medicine, Alfaisal University, Riyadh, Saudi Arabia

**Keywords:** Mitomycin C, Deep Sclerectomy, Congenital Glaucoma, Case report

## Abstract

**Introduction and importance:**

Congenital glaucoma poses a complex surgical challenge. Mitomycin-C (MMC) is frequently used to enhance surgical success, though the optimal method of application remains debated.

**Case presentation:**

We present a pediatric case of bilateral congenital glaucoma where two different MMC application methods were used: subconjunctival injection in one eye and sponge application in the other.

**Clinical discussion:**

Despite initial IOP reduction in both eyes, the eye treated with sponge application showed progressive bleb thinning and compromise. The subconjunctival injection resulted in a stable, diffuse bleb with better long-term outcomes.

**Conclusion:**

This case provides comparative intra-patient evidence favoring subconjunctival injection for improved bleb morphology and stability in pediatric glaucoma.

## Highlights

• First reported intra-patient comparison of MMC sponge vs. injection in pediatric DS
• Subconjunctival MMC injection led to more stable, diffuse bleb morphology
• MMC sponge application resulted in progressive bleb thinning, compromise and higher risk of late complications
• MMC delivery technique may influence long-term filtration success in children

## 1 Introduction

Glaucoma represents a heterogeneous group of optic neuropathies characterized by progressive damage to the optic nerve and visual field loss, ultimately leading to irreversible blindness if inadequately treated [1,2]. Among these, primary congenital glaucoma (PCG) is a rare but severe subtype, arising from abnormal anterior chamber angle development and typically presenting in infancy or early childhood [3,4]. The global incidence of PCG is estimated at 1 in 10,000–18,000 live births, though regional variations exist [5]. Clinical hallmarks include epiphora, photophobia, blepharospasm, and corneal changes (e.g., edema, Haab's striae). Without prompt IOP reduction, rapid and irreversible vision loss can occur [3,4].

Because elevated IOP is the primary modifiable risk factor in glaucoma pathogenesis [1], management focuses on reducing IOP to preserve optic nerve function. While topical medications and laser procedures can be effective in selected pediatric cases, surgical intervention is frequently required due to anatomical limitations and challenges in adherence to therapy [5].

First-line interventions for PCG include angle surgeries (goniotomy or trabeculotomy), particularly when the cornea remains sufficiently clear for visualization [6,7]. However, in advanced disease or when angle-based procedures fail, filtering operations such as trabeculectomy or deep sclerectomy (DS) are often necessary [8,9]. Trabeculectomy, originally popularized by Cairns, is a full-thickness filtration procedure, whereas DS is a non-penetrating technique designed to minimize early hypotony and bleb leaks while still achieving IOP reduction [8].

For refractory pediatric glaucoma, glaucoma drainage devices (GDDs) such as Baerveldt or Ahmed implants may be used to divert aqueous humor externally. In cases where conventional surgical approaches are insufficient, cyclodestructive procedures (e.g., diode laser cyclophotocoagulation) can be employed to ablate ciliary body tissue and reduce aqueous production [4,11].

When performing filtering surgeries, achieving a functional bleb is crucial for long-term IOP control [12,13]. An optimal bleb is diffuse, mildly elevated, minimally vascularized, and non-leaking, whereas cystic or highly vascular blebs increase the risk of leaks, hypotony, and bleb-related infections. Several factors influence bleb morphology, including surgical technique, patient-specific healing responses, and postoperative interventions such as needling or suture adjustments. [3,[12], [13], [14]].

Mitomycin C (MMC), a potent antimetabolite, is commonly used to inhibit fibroblast proliferation and minimize scarring in pediatric filtration surgeries [15,16]. Delivery methods include subconjunctival injection, which provide broader tissue exposure, and sponge application, which localizes MMC to the surgical site [15,16]. Because children often require multiple surgical procedures, balancing wound modulation with conjunctival preservation is crucial [4]. Anterior segment optical coherence tomography (AS-OCT) has enhanced bleb evaluation, allowing early detection of micro-leaks, encapsulation, or thinning [13,17].

Recent studies suggest that subconjunctival MMC injection results in more diffuse, stable blebs compared to sponge application, which may lead to focal tissue necrosis and inconsistent drug delivery (18–21). This case report evaluates the effects of MMC delivery methods on conjunctival bleb morphology and long-term surgical success in a pediatric patient with congenital glaucoma.

This case report has been reported in line with the SCARE 2025 guidelines [25].

## 2 Case Presentation

A 2-year-old female with congenital glaucoma underwent bilateral deep sclerectomy with MMC (0.2 mg/ml via sponge for 2 min) which was performed by the same surgeon during infancy. Initially, blebs were diffused and functional, IOP was controlled without adjunctive medications, and the patient was prescribed corrective glasses for progressive myopia. However, serial follow-ups revealed bleb fibrosis and flatting, optic disc cupping progression and recurrent IOP elevation, necessitating topical Azarga (brinzolamide/timolol) twice daily in both eyes.

**Fig. 1.1:**
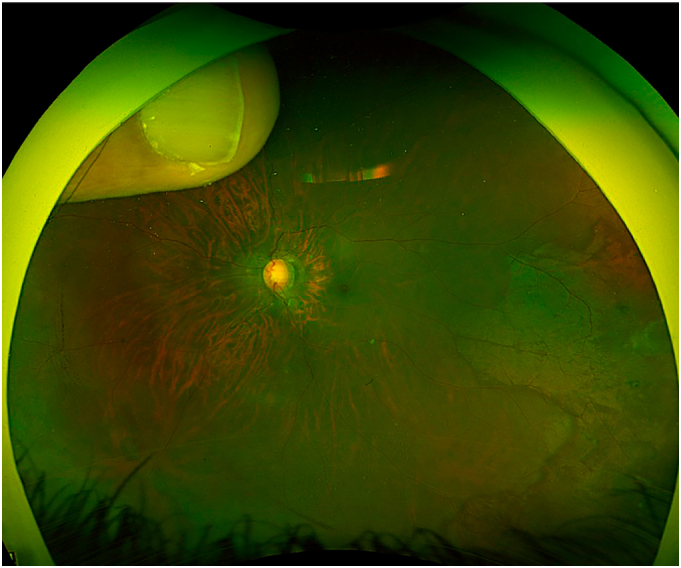
Wide-field fundus photograph of the left eye demonstrating optic disc cupping, consistent with glaucoma progression prior to repeat deep sclerectomy.

**Fig. 1.2:**
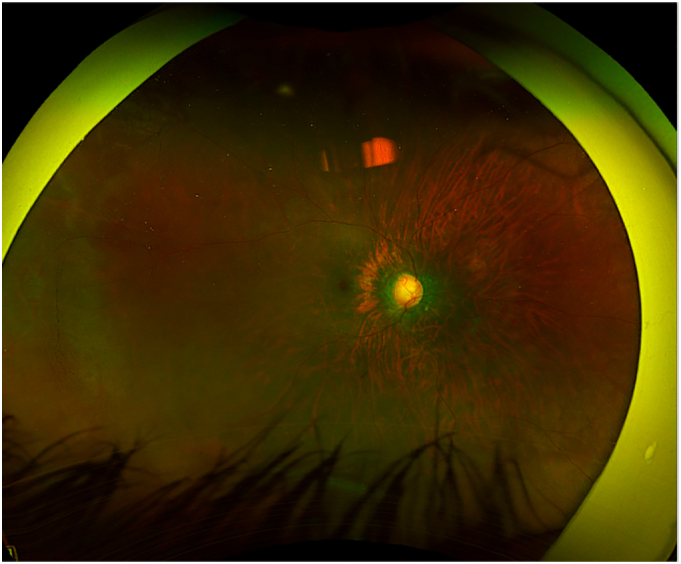
Wide-field fundus photograph of the right eye demonstrating optic disc cupping, consistent with glaucoma progression prior to repeat deep sclerectomy.

By age 3, poor adherence to therapy led to worsening cup-to-disc ratios (0.8 OD, 0.9 OS) and persistently elevated IOP, reaching 24 mmHg in OS. Apraclonidine (Iopidine) was added, but surgical reintervention became necessary. (See Fig. 1.1, Fig. 1.2.)

At age 5, the patient underwent repeat DS with MMC in OS (February 2024). Intraoperatively, MMC (0.4 mg/ml via a sponge for 3 min) beneath the scleral flap then washed thoroughly. Postoperatively, IOP decreased to 8 mmHg, but subsequent visits revealed progressive bleb thinning and compromise. Three months after the surgery, OD exhibited an IOP spike to 32 mmHg, necessitating repeat DS with MMC. This time, MMC (0.1 ml of 0.4 mg/ml via subconjunctival injection for 3 min) in the subconjunctival space in the superior temporal quadrant after the superficial scleral flap and before the deep one then washed thoroughly. Postoperative IOP dropped to 4 mmHg, and the bleb remained stable with no fragility.

During the five to six months follow-ups after the repeated DC with MMC, OD (subconjunctival MMC eye) exhibited a thicker, more stable bleb, with IOP sustained at 12–13 mmHg. OS (sponge-applied MMC eye), Despite an initial IOP of 10 mmHg, the bleb showed progressive thinning and morphological compromise with a higher risk of encapsulation and bleb failure, without hypotony-related complications such as shallow anterior chamber, choroidal folds, maculopathy, or bleb leak. The patient was managed with a gradual topical steroid taper and close monitoring.

**Table:** Timeline.

Age	Event
2 years	Initial DS with MMC sponge (OU)
3 years	Poor adherence, worsening IOP
5 years	Repeat DS (OS: sponge-applied MMC eye), later (OD: subconjunctival MMC eye)
5.5 years	Follow-up: stable bleb in OD, compromised bleb in OS

## 3 Discussion

Managing congenital glaucoma requires long-term planning, particularly in pediatric patients who often undergo multiple surgical interventions [22]. This case underscores the differences in bleb morphology and surgical outcomes associated with MMC delivery methods.

MMC remains a cornerstone in glaucoma surgery, enhancing surgical success by inhibiting fibroblast proliferation and reducing scarring [23]. In this case, subconjunctival MMC injection provided more stable bleb morphology and a lower risk of encapsulation compared to sponge application. In contrast, the absorbent capacity of sponges varies, leading to inconsistent MMC delivery and potential focal tissue necrosis [[18], [19], [20], [21]]. Studies indicate that injection-based MMC application ensures a broader treatment area and more predictable drug diffusion, leading to reduced scarring and improved surgical outcomes [22,23].

**Fig. 2:**
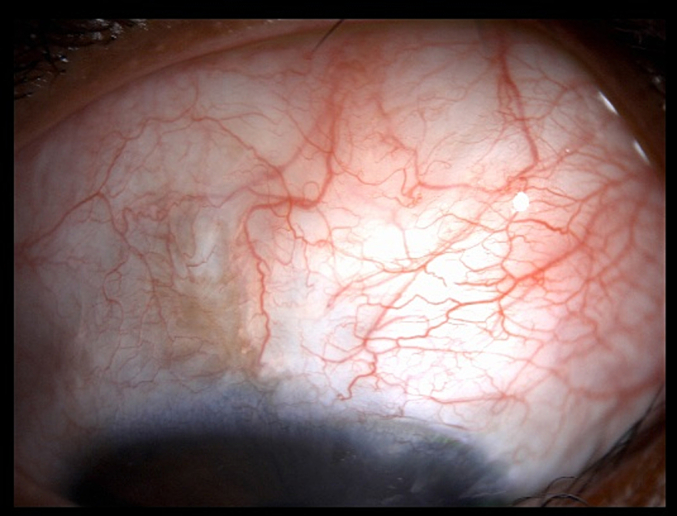
Slit lamp photo of the right eye (OD) post-subconjunctival MMC injection, showing a thick, diffuse bleb with a healthy conjunctival surface.

**Fig. 3:**
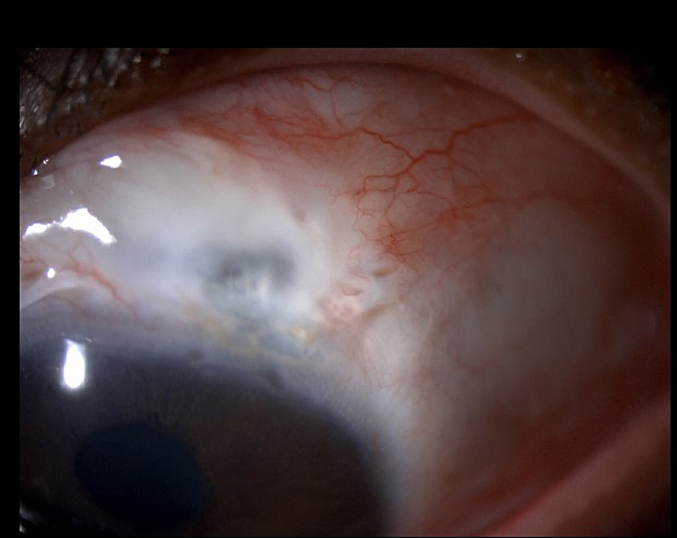
Slit lamp photo of the left eye (OS) post-MMC sponge application, demonstrating progressive conjunctival thinning and bleb compromise.

In our patient, OD (subconjunctival MMC eye) demonstrated a more stable bleb with no marked conjunctival thinning, even though IOP decreased to 4 mmHg (Fig. 2). Conversely, OS (sponge-applied MMC eye) achieved low postoperative IOP (8 mmHg) but developed progressive conjunctival compromise over time (Fig. 3). While previous reports have suggested sponge application may reduce complications such as bleb leakage) [24]. Our findings align with emerging evidence favoring subconjunctival injection in pediatric glaucoma surgery. A limitation of this report is the relatively short follow-up (6–7 months), as bleb morphology and function in pediatric glaucoma may change over several years. Long-term follow-ups and larger studies are needed to further validate these advantages and refine surgical techniques. (See Fig. 1.1, Fig. 1.2.)

## 4 Conclusion

This case highlights the impact of MMC application techniques on surgical outcomes in pediatric congenital glaucoma. While both approaches successfully reduced IOP, subconjunctival injection produced a more stable bleb with minimal thinning, whereas sponge application led to progressive conjunctival compromise. These findings suggest that subconjunctival MMC injection may be the superior technique for pediatric glaucoma surgery, offering better long-term filtration and bleb stability. Further research should explore this approach to optimize surgical success in pediatric patients.

## Consent

Written informed consent was obtained from the patient's guardian for publication of this case and accompanying images. A copy is available upon request by the Editor-in-Chief.

## Patient perspective

The patient's guardian expressed satisfaction with the outcome and was particularly reassured by the better recovery and appearance of the right eye.

## Ethical approval

Ethical approval for this study (IRB Approval No. RD/26001/IRB/0203-25) was granted by the Institutional Review Board at King Khaled Eye Specialist Hospital, Riyadh, Saudi Arabia on 27 February 2025.

## Funding

No funding received for this case report.

## Author contribution

**Mohammed A. Halawani:** Conceptualization, data collection, analysis, manuscript drafting

**Dania A. Bamefleh:** Case idea originator, consultant supervision, critical revision

**Saad Alosaimi:** Literature review, writing support, figure editing

**Raghad Alonazi:** Data interpretation, reference management, manuscript editing

## Guarantor

Dr. Mohammed Halawani and Dr. Dania Bamefleh accepts full responsibility for the integrity of the work, access to data, and decision to publish.

## Research registration number

Not applicable

## Conflict of interest statement

The authors declare that there are no conflicts of interest related to this case report.
